# (*RS*/*SR*)-2-Oxo-4-phenyl­azetidin-3-yl acetate

**DOI:** 10.1107/S1600536809038860

**Published:** 2009-09-30

**Authors:** Yangjun Li

**Affiliations:** a School of Information and Communication Engineering, North University of China, Taiyuan 030051, People’s Republic of China

## Abstract

In the title compound, C_11_H_11_NO_3_, a modified synthetic acetate derivative, the four memebered β-lactam ring is roughly planar, with a maximum deviation of 0.21 (3) Å, and makes a dihedral angle of 81.46 (14)° with the phenyl ring. In the crystal, a single N—H⋯O hydrogen bond links mol­ecules into a chain parallel to the *a* axis and thus stabilizes the structure. Although the absolute configuration could not be reliably determined, the compound corresponds to the diasteroisomer (*RS*/*SR*)

## Related literature

For properties of lactams, see: Selvanayagam *et al.* (2005 ▶); Deschamps *et al.* (2003 ▶); Kanazawa *et al.* (1993 ▶). For a related structure, see: Akkurt *et al.* (2007 ▶).
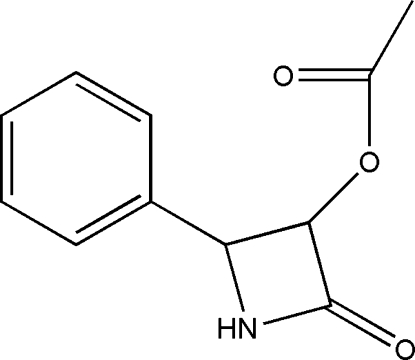


## Experimental

### 

#### Crystal data

C_11_H_11_NO_3_
*M* *_r_* = 205.21
Orthorhombic, 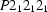

*a* = 5.940 (4) Å
*b* = 8.198 (4) Å
*c* = 20.896 (13) Å
*V* = 1017.6 (11) Å^3^
*Z* = 4
Mo *K*α radiation
μ = 0.10 mm^−1^
*T* = 298 K
0.21 × 0.16 × 0.10 mm

#### Data collection

Bruker APEXII area-detector diffractometer
Absorption correction: multi-scan (*SADABS*; Bruker, 2005 ▶) *T*_min_ = 0.980, *T*_max_ = 0.990
1899 measured reflections
1126 independent reflections
853 reflections with *I* > 2σ(*I*)
*R*_int_ = 0.027

#### Refinement

*R*[*F* ^2^ > 2σ(*F* ^2^)] = 0.041
*wR*(*F* ^2^) = 0.125
*S* = 1.17
1126 reflections
137 parameters
H-atom parameters constrained
Δρ_max_ = 0.17 e Å^−3^
Δρ_min_ = −0.19 e Å^−3^

### 

Data collection: *APEX2* (Bruker, 2005 ▶); cell refinement: *SAINT* (Bruker, 2005 ▶); data reduction: *SAINT* ; program(s) used to solve structure: *SHELXS97* (Sheldrick, 2008 ▶); program(s) used to refine structure: *SHELXL97* (Sheldrick, 2008 ▶); molecular graphics: *ORTEPIII* (Burnett & Johnson, 1996 ▶), *ORTEP-3 for Windows* (Farrugia, 1997 ▶) and *PLATON* (Spek, 2009 ▶); software used to prepare material for publication: *SHELXL97*.

## Supplementary Material

Crystal structure: contains datablocks I, global. DOI: 10.1107/S1600536809038860/dn2490sup1.cif

Structure factors: contains datablocks I. DOI: 10.1107/S1600536809038860/dn2490Isup2.hkl

Additional supplementary materials:  crystallographic information; 3D view; checkCIF report

## Figures and Tables

**Table 1:** Hydrogen-bond geometry (Å, °)

*D*—H⋯*A*	*D*—H	H⋯*A*	*D*⋯*A*	*D*—H⋯*A*
N1—H1*A*⋯O1^i^	0.86	2.11	2.943 (3)	162

Symmetry code: (i) 
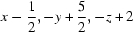
.

**Fig. 1.**
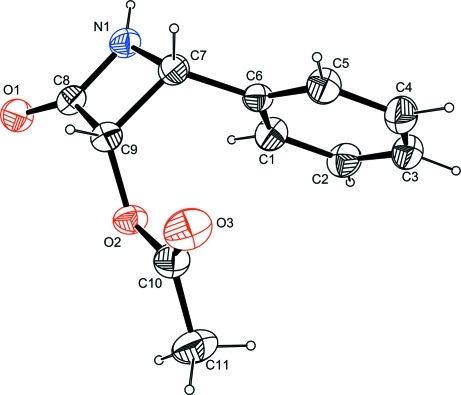
Molecular view of (I) with the atom-labeling scheme. Ellipsoids are drawn at the the 30% probability level. H atoms are shown as spheres of arbitrary radii.

**Fig. 2.**
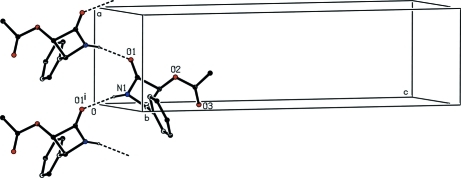
Partial packing view showing the formation of the chain parallel to the a axis. H atoms not involved in hydrogen bondings have been omitted for clarity. [Symmetry code: (i) x-1/2, -y+5/2, -z+2]

## supplementary crystallographic information

### Comment

Recently, lactams have attracted much attention because they are convenient intermediates for semi-synthesis of the antitumour drug Taxol and other bioactive analogues (Kanazawa *et al.*, 1993). Furthermore, the lactam ring (azetidin-2-one) is considered a general 'lead structure' for the design of new inhibitors of enzymes containing a serine nucleophile in the active site (Deschamps *et al.*, 2003). In an attempt to form a Zn(II) complex with title compound, we adventitiously formed the title compound (I) and its crystal structure is determined herein.

The molecular structure of (I) is illustrated in Fig. 1. It is very similar to the related 4-(4-Nitrophenyl)-3-phenoxyazetidin-2-one (Akkurt *et al.*, 2007). The geometry of the β-lactam ring is is planar, with a maximum deviation of 0.21 (3)° for atom N1. It makes dihedral angles of 81.46 (14)° with its phenyl substituent. The lactam ting is also comparable with a related reported structure (Selvanayagam *et al.*, 2005). Although the absolute configuration couldn't be reliably determined, the compound correspond to the diasteroisomer (RS/SR).

Intermolecular N-H···O hydrogen bonds form a zig-zag like chain parallel to the a axis and thus stabilize the structure. (Table 1, Figure 2).

### Experimental

The title compound was obtained by direct mixing of equimolar (28mg, 0.1mmol) Zn(OAC)_2_.6H_2_O of water solution (8mL) and 2-Oxo-4-phenylazetidin-3-yl acetate (21mg, 0.1mmol), and CH_3_CN and CH_3_CH_2_OH solutions (5mL). using slow evaporation of the solvent at room temperature over a period of about two weeks.

### Refinement

In the absence of significant anomalous scattering, the absolute configuration could not be reliably determined and then the Friedel pairs were merged and any references to the Flack parameter were removed.

All H atoms were placed in calculated positions (C-H = 0.93 (aromatic), N-H=0.86, or 0.96 Å (methyl)) refined using a riding model, with U_iso_(H) = 1.2U_eq_(C)(aromatic), U_iso_(H) = 1.5U_eq_(C) (methyl).

### Crystal data

**Table:**

C_11_H_11_NO_3_	*F*(000) = 432
*M**_r_* = 205.21	*D*_x_ = 1.340 Mg m^−^^3^
Orthorhombic, *P*2_1_2_1_2_1_	Mo *K*α radiation, λ = 0.71073 Å
Hall symbol: P 2ac 2ab	Cell parameters from 1899 reflections
*a* = 5.940 (4) Å	θ = 2.0–25.5°
*b* = 8.198 (4) Å	µ = 0.10 mm^−^^1^
*c* = 20.896 (13) Å	*T* = 298 K
*V* = 1017.6 (11) Å^3^	Block, colorless
*Z* = 4	0.21 × 0.16 × 0.10 mm

### Data collection

**Table:**

Bruker APEXII area-detector diffractometer	1126 independent reflections
Radiation source: fine-focus sealed tube	853 reflections with *I* > 2σ(*I*)
graphite	*R*_int_ = 0.027
φ and ω scans	θ_max_ = 25.5°, θ_min_ = 2.0°
Absorption correction: multi-scan (*SADABS*; Bruker, 2005)	*h* = −7→7
*T*_min_ = 0.980, *T*_max_ = 0.990	*k* = 0→9
1899 measured reflections	*l* = −25→0

### Refinement

**Table:**

Refinement on *F*^2^	Primary atom site location: structure-invariant direct methods
Least-squares matrix: full	Secondary atom site location: difference Fourier map
*R*[*F*^2^ > 2σ(*F*^2^)] = 0.041	Hydrogen site location: inferred from neighbouring sites
*wR*(*F*^2^) = 0.125	H-atom parameters constrained
*S* = 1.17	*w* = 1/[σ^2^(*F*_o_^2^) + (0.0728*P*)^2^] where *P* = (*F*_o_^2^ + 2*F*_c_^2^)/3
1126 reflections	(Δ/σ)_max_ = 0.001
137 parameters	Δρ_max_ = 0.17 e Å^−^^3^
0 restraints	Δρ_min_ = −0.19 e Å^−^^3^

### Special details

**Table:**

Geometry. All esds (except the esd in the dihedral angle between two l.s. planes) are estimated using the full covariance matrix. The cell esds are taken into account individually in the estimation of esds in distances, angles and torsion angles; correlations between esds in cell parameters are only used when they are defined by crystal symmetry. An approximate (isotropic) treatment of cell esds is used for estimating esds involving l.s. planes.
Refinement. Refinement of F^2^ against ALL reflections. The weighted R-factor wR and goodness of fit S are based on F^2^, conventional R-factors R are based on F, with F set to zero for negative F^2^. The threshold expression of F^2^ > 2sigma(F^2^) is used only for calculating R-factors(gt) etc. and is not relevant to the choice of reflections for refinement. R-factors based on F^2^ are statistically about twice as large as those based on F, and R- factors based on ALL data will be even larger.

### Fractional atomic coordinates and isotropic or equivalent isotropic displacement parameters (Å^2^)

**Table:**

	*x*	*y*	*z*	*U*_iso_*/*U*_eq_	
C1	0.6268 (6)	0.7783 (4)	0.93447 (16)	0.0536 (9)	
H1	0.7589	0.8249	0.9498	0.064*	
C2	0.5969 (7)	0.6098 (4)	0.93779 (16)	0.0598 (10)	
H2	0.7093	0.5443	0.9551	0.072*	
C3	0.4038 (7)	0.5413 (4)	0.91576 (16)	0.0613 (10)	
H3	0.3853	0.4288	0.9176	0.074*	
C4	0.2362 (7)	0.6368 (4)	0.89092 (16)	0.0624 (10)	
H4	0.1036	0.5894	0.8763	0.075*	
C5	0.2647 (6)	0.8040 (4)	0.88758 (14)	0.0543 (9)	
H5	0.1502	0.8688	0.8710	0.065*	
C6	0.4617 (5)	0.8759 (4)	0.90866 (13)	0.0412 (7)	
C7	0.4897 (6)	1.0577 (3)	0.90075 (14)	0.0449 (7)	
H7	0.3451	1.1122	0.8935	0.054*	
C8	0.7926 (6)	1.1852 (3)	0.91243 (13)	0.0434 (7)	
C9	0.6733 (5)	1.1182 (3)	0.85319 (13)	0.0418 (7)	
H9	0.6188	1.2043	0.8246	0.050*	
C10	0.7031 (7)	0.9236 (4)	0.77046 (14)	0.0523 (9)	
C11	0.8572 (7)	0.7982 (4)	0.74195 (15)	0.0747 (12)	
H11A	0.8191	0.6924	0.7584	0.112*	
H11B	1.0101	0.8237	0.7530	0.112*	
H11C	0.8409	0.7984	0.6962	0.112*	
N1	0.6232 (5)	1.1404 (3)	0.95028 (11)	0.0480 (7)	
H1A	0.5996	1.1550	0.9905	0.058*	
O1	0.9694 (4)	1.2566 (3)	0.92206 (9)	0.0555 (6)	
O2	0.8050 (4)	0.9987 (2)	0.82088 (8)	0.0468 (6)	
O3	0.5181 (5)	0.9564 (3)	0.75310 (12)	0.0712 (7)	

### Atomic displacement parameters (Å^2^)

**Table:**

	*U*^11^	*U*^22^	*U*^33^	*U*^12^	*U*^13^	*U*^23^
C1	0.048 (2)	0.0518 (18)	0.0607 (19)	0.0040 (16)	−0.0083 (17)	−0.0020 (16)
C2	0.067 (3)	0.0467 (18)	0.065 (2)	0.0129 (18)	−0.001 (2)	0.0082 (16)
C3	0.072 (3)	0.0455 (17)	0.066 (2)	−0.0057 (19)	0.009 (2)	−0.0021 (16)
C4	0.057 (2)	0.0561 (19)	0.074 (2)	−0.0084 (18)	−0.003 (2)	−0.0076 (17)
C5	0.050 (2)	0.0534 (18)	0.0593 (19)	0.0017 (17)	−0.0051 (17)	−0.0001 (15)
C6	0.0416 (19)	0.0429 (14)	0.0391 (14)	0.0017 (15)	0.0034 (14)	−0.0025 (12)
C7	0.0427 (19)	0.0433 (15)	0.0486 (15)	0.0005 (15)	0.0000 (15)	−0.0026 (12)
C8	0.049 (2)	0.0366 (14)	0.0448 (16)	0.0036 (15)	−0.0017 (16)	−0.0025 (12)
C9	0.0434 (19)	0.0418 (13)	0.0403 (14)	0.0038 (15)	−0.0018 (14)	−0.0026 (13)
C10	0.061 (2)	0.0573 (18)	0.0385 (15)	−0.0031 (18)	−0.0030 (16)	−0.0029 (13)
C11	0.073 (3)	0.083 (2)	0.068 (2)	0.009 (2)	−0.002 (2)	−0.030 (2)
N1	0.0590 (18)	0.0466 (13)	0.0383 (12)	−0.0024 (13)	0.0040 (13)	−0.0064 (11)
O1	0.0506 (15)	0.0620 (13)	0.0540 (12)	−0.0106 (12)	−0.0019 (11)	−0.0112 (10)
O2	0.0450 (13)	0.0540 (11)	0.0415 (11)	0.0019 (12)	−0.0005 (9)	−0.0109 (9)
O3	0.0721 (18)	0.0842 (16)	0.0572 (12)	0.0093 (16)	−0.0186 (13)	−0.0125 (12)

### Geometric parameters (Å, °)

**Table:**

C1—C6	1.376 (4)	C7—H7	0.9800
C1—C2	1.395 (4)	C8—O1	1.219 (4)
C1—H1	0.9300	C8—N1	1.332 (4)
C2—C3	1.357 (5)	C8—C9	1.529 (4)
C2—H2	0.9300	C9—O2	1.424 (3)
C3—C4	1.369 (5)	C9—H9	0.9800
C3—H3	0.9300	C10—O3	1.188 (4)
C4—C5	1.383 (4)	C10—O2	1.362 (4)
C4—H4	0.9300	C10—C11	1.500 (5)
C5—C6	1.382 (4)	C11—H11A	0.9600
C5—H5	0.9300	C11—H11B	0.9600
C6—C7	1.509 (4)	C11—H11C	0.9600
C7—N1	1.469 (4)	N1—H1A	0.8600
C7—C9	1.556 (4)		
			
C6—C1—C2	120.3 (4)	C9—C7—H7	111.8
C6—C1—H1	119.8	O1—C8—N1	133.3 (3)
C2—C1—H1	119.8	O1—C8—C9	134.9 (3)
C3—C2—C1	120.0 (4)	N1—C8—C9	91.8 (2)
C3—C2—H2	120.0	O2—C9—C8	112.1 (2)
C1—C2—H2	120.0	O2—C9—C7	117.9 (2)
C2—C3—C4	120.4 (3)	C8—C9—C7	85.5 (2)
C2—C3—H3	119.8	O2—C9—H9	112.8
C4—C3—H3	119.8	C8—C9—H9	112.8
C3—C4—C5	119.8 (4)	C7—C9—H9	112.8
C3—C4—H4	120.1	O3—C10—O2	123.0 (3)
C5—C4—H4	120.1	O3—C10—C11	126.7 (3)
C6—C5—C4	120.7 (3)	O2—C10—C11	110.2 (3)
C6—C5—H5	119.7	C10—C11—H11A	109.5
C4—C5—H5	119.7	C10—C11—H11B	109.5
C1—C6—C5	118.7 (3)	H11A—C11—H11B	109.5
C1—C6—C7	122.6 (3)	C10—C11—H11C	109.5
C5—C6—C7	118.7 (3)	H11A—C11—H11C	109.5
N1—C7—C6	116.0 (3)	H11B—C11—H11C	109.5
N1—C7—C9	85.7 (2)	C8—N1—C7	96.7 (2)
C6—C7—C9	117.5 (2)	C8—N1—H1A	131.6
N1—C7—H7	111.8	C7—N1—H1A	131.6
C6—C7—H7	111.8	C10—O2—C9	115.7 (2)

### Hydrogen-bond geometry (Å, °)

**Table:**

*D*—H···*A*	*D*—H	H···*A*	*D*···*A*	*D*—H···*A*
N1—H1A···O1^i^	0.86	2.11	2.943 (3)	162

Symmetry codes: (i) *x*−1/2, −*y*+5/2, −*z*+2.
